# Spatial and temporal profile of cisplatin delivery by ultrasound-assisted intravesical chemotherapy in a bladder cancer model

**DOI:** 10.1371/journal.pone.0188093

**Published:** 2017-11-30

**Authors:** Noboru Sasaki, Kazuhiro Ishi, Nobuki Kudo, Shouta M. M. Nakayama, Kensuke Nakamura, Keitaro Morishita, Hiroshi Ohta, Mayumi Ishizuka, Mitsuyoshi Takiguchi

**Affiliations:** 1 Department of Clinical Sciences, Faculty of Veterinary Medicine, Hokkaido University, Sapporo, Japan; 2 Division of Bioengineering and Bioinformatics, Graduate School of Information Science and Technology, Hokkaido University, Sapporo, Japan; 3 Department of Environmental Veterinary Sciences, Faculty of Veterinary Medicine, Hokkaido University, Sapporo, Japan; 4 Veterinary Teaching Hospital, Faculty of Veterinary Medicine, Hokkaido University, Sapporo, Japan; Nanjing University, CHINA

## Abstract

Non-muscle invasive bladder cancer is one of the most common tumors of the urinary tract. Despite the current multimodal therapy, recurrence and progression of disease have been challenging problems. We hereby introduced a new approach, ultrasound-assisted intravesical chemotherapy, intravesical instillation of chemotherapeutic agents and microbubbles followed by ultrasound exposure. We investigated the feasibility of the treatment for non-muscle invasive bladder cancer. In order to evaluate intracellular delivery and cytotoxic effect as a function to the thickness, we performed all experiments using a bladder cancer mimicking 3D culture model. Ultrasound-triggered microbubble cavitation increased both the intracellular platinum concentration and the cytotoxic effect of cisplatin at the thickness of 70 and 122 μm of the culture model. The duration of enhanced cytotoxic effect of cisplatin by ultrasound-triggered microbubble cavitation was approximately 1 hr. Based on the distance and duration of delivery, we further tested the feasibility of repetition of the treatment. Triple treatment increased the effective distance by 1.6-fold. Our results clearly showed spatial and temporal profile of delivery by ultrasound-triggered microbubble cavitation in a tumor-mimicking structure. Furthermore, we demonstrated that the increase in intracellular concentration results in the enhancement of the cytotoxic effect in a structure with the certain thickness. Repetition of ultrasound exposure would be treatment of choice in future clinical application. Our results suggest ultrasound-triggered microbubble cavitation can be repeatable and is promising for the local control of non-muscle invasive bladder cancer.

## Introduction

Bladder cancer is the ninth most common cancer in the world, and almost 70% are non-muscle invasive bladder cancers (NMIBC) at initial diagnosis [1]. The standard therapy is transurethral resection of visible tumors, followed by intravesical chemotherapy or Bacillus Calmette-Guérin (BCG) immunotherapy [2]. Although BCG vaccine is the most effective agent to reduce recurrences and delay disease progression, 50–70% of patients relapse within 5 years and up to 30% progress to muscle invasive cancer [3,4]. Radical cystectomy remains the standard therapy after the BCG failure, however radical cystectomy causes several adverse effects and has impacts on quality of life [5]. Hence, new and more effective therapeutic strategies are clearly warranted for local control of NMIBC with well tolerability.

Ultrasound (US)-triggered microbubble cavitation, also known as sonoporation, is minimally invasive drug delivery technology. Oscillation and collapse of gas-filled microbubbles upon ultrasound exposure transiently increase the cell membrane permeability, which results in uptake of extracellular molecules by cells. Bladder cancer would be one of the most promising targets for drug delivery by US-triggered microbubble cavitation. Intravesical instillation enables microbubbles to directly attach to the surface of bladder. Since NIMBC locates urothelium/lamina propria, US-triggered microbubble cavitation in bladder would be effective with high tumor specificity. Both drug concentration and exposure time determine the efficacy of intravesical chemotherapy [6]. It is critical for developing clinical effective US-assisted intravesical chemotherapy to achieve efficient drug penetration in tumor tissue and to increase intracellular drug concentration. In addition, duration of drug delivery may influence clinical protocols of US-assisted intravesical chemotherapy in future. Previous studies showed that US-triggered microbubble cavitation enhanced penetration of chromophores into tissue [7–9] and had certain duration of increased cell membrane permeability [10–13]. To our knowledge, however, it has not fully been elucidated that US-triggered microbubble cavitation enhances cellular uptake of chemotherapeutic agents in tissue or tissue-mimicking model. Moreover, the duration of enhanced cytotoxic effect of chemotherapeutic agents by single US-triggered microbubble cavitation is obscure. From these perspectives, we evaluated a spatial and temporal profile of cisplatin delivery by US-triggered microbubble cavitation using a bladder cancer mimicking three-dimensional (3D) culture model. Cisplatin was evaluated as one of the intravesical chemotherapeutic agents in clinic [14,15] and preclinical research [16,17]. We assessed the intracellular concentration and the cytotoxic effect of cisplatin as a function of the thickness of the 3D model. In addition to the penetration profile, the intervals between sonication and cisplatin exposure was investigated using the 3D model.

## Materials and methods

### Cells and the 3D model

Human urinary bladder transitional cell carcinoma cells (UM-UC-3; American Type Culture Collection, Manassas, VA, USA) were maintained in alfa Minimum Essential Medium (MEM-alfa; Sigma-Aldrich, St. Louis, MO, USA) supplemented with 10% fetal bovine serum. For the 3D model, 2 x 10^7^ cells/mL of UM-UC-3 were embedded in collagen-I (Collagen I High concentration, Rat tail; Corning Inc., Belford, MA, USA) according to the previous study [18]. The collagen solution consisted of 10x MEM-alfa (Sigma-Aldrich), a buffer solution, additional 1 M NaOH (to adjust pH). The final collagen concentration was 3 mg/mL. The cell containing solution was added to 35 mm dish (Nunclon™Delta surface; Thermo Fisher Scientific, Suzhou, China) at a seeding volume of 50, 100, 150, or 200 μL. The solution was incubated to solidify for 30 min at 37°C. UM-UC-3 cells in the 3D model were cultured for 3 days before experiments. In order to measure the thickness of the 3D model, fluorescent microscopic images of cells in z-stuck were obtained by BZ-9000 (Keyence, Osaka, Japan). Cells were incubated with 2 μM Calcein-AM (Wako Pure Chemicals) for 30 min at 37°C prior to the imaging.

### Cisplatin and microbubbles

Cisplatin (Wako Pure Chemical Industries Ltd., Osaka, Japan) was dissolved in saline at a concentration of 1 mg/mL (stock solution). An US contrast agent (Sonazoid™; DaiichiSankyo, Tokyo, Japan) was used as microbubbles. The contrast agent is comprised of a phospholipid shell encapsulating perfluorobutane gas. Sonazoid™ was reconstituted in 2 mL of sterilized distilled water according to the manufacturer’s instructions, which contains 1.2 x 10^9^ microbubbles/mL with an average diameter of 3.2 μm [19,20].

### Ultrasound setup (Fig 1)

Ultrasound experiments were performed with a single-element flat-faced transducer (Fuji Ceramic, Shizuoka, Japan). A sinusoidal electrical signal was generated by an arbitrary function generator (AFG1022; Techtoronics, Tokyo, Japan), which was amplified by a power amplifier (T145-5315A; Samway, Shizuoka, Japan). The diameter of the transducer was 50 mm. The theoretical distance of the acoustic near field is 480 mm (D_T_^2^/4λ, where D_T_ is the diameter of the transducer and λ is the wavelength). Ultrasound parameters were as follows; 1 MHz center frequency, 50% duty cycle (500 μsec pulse width, 1 kHz pulse repetition frequency). These parameters were used for ultrasound-mediated cytotoxic agents delivery *in vitro* and *in vivo* [21–24]. In our set up, ultrasound intensity was 90 mW/cm^2^, which corresponds to 0.13 MPa peak-to-peak pressure. The spatial-average temporal-average US intensity was determined by the measurement of acoustic radiation force using a microbalance. The pressure of the acoustic wave was measured in a separate setup using a calibrated hydrophone (HMB-0500; Onda, Sunnyvale, CA, USA) at 5 cm distance from the transducer.

**Fig 1:**
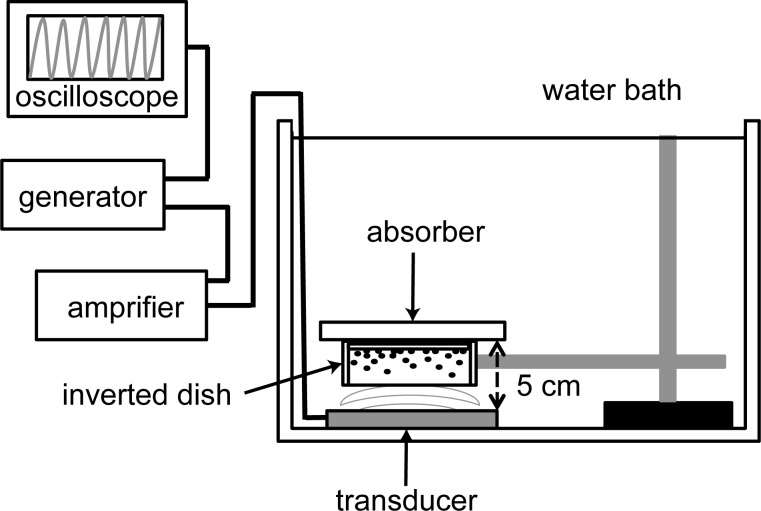
Schematic illustration of the ultrasound setup. A single-element flat-faced transducer, the diameter of 50 mm, was driven by an arbitrary function generator and a power amplifier. Inverted 35 mm dish was placed 5 cm above the ultrasound transducer in a water bath. An absorber was placed on the inverted dish to prevent generation of standing waves.

### Ultrasound exposure

After culturing for 3 days, cells in the 3D model were exposed to ultrasound. For the experiment, 171 μL of cisplatin stock solution was mixed with MEM-alfa to make 28.5 mL of an exposure medium. The cisplatin concentration of the exposure medium was 6 μg/mL. The exposure medium was divided into three aliquots (9.5 mL) because 9.5 mL was sufficient to completely fill the 35 mm dish. Just before the treatment, 190 μL of Sonazoid™ was added to the exposure medium. The dish was covered with Parafilm M™ (Bemis Company Inc., Neenah, IL, USA) in order to prevent from air entering the medium. To minimize an excess diffusion of cisplatin into the collagen gel, the covered dish was immediately inverted and exposed to US for 1 min in a water bath. The bottom of the inverted dish was located 5 cm above the transducer. During sonication, an absorber was placed on the inverted dish to prevent generation of standing waves.

### Spatial profile of delivery (S1 Fig)

#### Platinum quantification

The exposure medium was removed immediately after the sonication, and the surface of the 3D model and the dish were rinsed once with 1 mL of PBS. The 3D culture was incubated for 30 min at 37°C with 1 mL of MEM-alfa containing 0.02% collagenase (Wako Pure Chemical). The dissolved solution was centrifuged at 12,000 rpm for 10sec, and 0.8 mL of supernatant was transferred to a new tube. Cell pellet was washed twice with 1 mL of PBS. The cell pellet and an aliquot of the supernatant were digested in 60% nitric acid for longer than 24 hr. The details of the sample pretreatment were shown in “S1 File”. Platinum (Pt) concentration in the cell pellet and the supernatant measured using an inductively coupled plasma mass spectrometry (Agilent 7700x ICP-MS; Agilent Technologies, Tokyo, Japan). The Pt concentration obtained with ICP-MS was in the range of 1–100 ppt (parts per trillion, 10^−12^) and was adjusted by a dilution factor for analysis.

#### Cell viability

The exposure medium was changed to the culture medium (i.e. cisplatin-free) after treatment. The cells were cultured in the 3D model for more 4 days. Thereafter, the 3D culture was incubated for 30 min at 37°C with 1 mL MEM-alfa containing 0.02% collagenase. The dissolved solution was centrifuged at 12,000 rpm for 10sec, and cell pellet was suspended in 1 mL of PBS. The number of viable cells was counted manually with the trypan-blue dye exclusion method. The cell viability was calculated as follows; the cell viability (%) = the number of cells in treatment group/the number of cells in non-treated group.

### Temporal profile of delivery (S1 Fig)

For this experiment, 100 μL of the cell containing solution was seeded to the 35 mm dish. Culturing for 3 days, 9.5 mL of MEM-alfa containing only 190 μL of Sonazoid™ (i.e. cisplatin-free) was added to the dish. Covered by Parafilm M™, the dish was inverted and exposed to US for 1 min in the water bath. The medium was changed to 2 mL of MEM-alfa containing 6 μg/mL cisplatin at different time points after the ultrasound exposure. The interval was 0 (right after), 0.5 hr, 1 hr, or 2 hr after the sonication. The dish was placed in the water bath for another 1 min without the ultrasound exposure. The viable cells were counted 4 days after the treatment as stated in “Cell viability”.

### Multiple treatments (S1 Fig)

For this experiment, 200 μL of the cell containing solution was seeded to the 35 mm dish. The cells were exposed to US three times at the interval of 30 min in the presence of 6 μg/mL cisplatin and 190 μL Sonazoid™. After each ultrasound exposure, cells were incubated for 30 min at 37°C with the cisplatin-free medium. The viable cells were counted 4 days after the treatment as stated in “Cell viability”.

### Statistics

Data were presented as mean ± standard deviation. Statistical analysis was performed using a computer program JMP Pro12 (SAS Institute Inc., Cary, NC, USA). Differences among groups were tested using analysis of variance with the Turkey’s post-hoc test and were considered statistically significant if the *p*-value was lower than < 0.05.

## Results

### The 3D model

The thickness of the 3D culture at the seeding volume of 50, 100, 150, and 200 μL was 70 ± 2.5, 122 ± 8.3, 153 ± 9.0, and 196 ± 10 µm, respectively (n = 3). The cell number of each volume was 1.3 ± 0.2 × 10^6^, 1.7 ± 0.4 × 10^6^, 2.0 ± 0.6 × 10^6^, and 3.1 ± 0.5 × 10^6^ cells, respectively (n = 3). Because the purpose of this study is to evaluate cisplatin delivery as a function of the thickness, results in following sections were shown in the thickness of the 3D model. S2 Fig shows an example of fluorescent microscopic images of the 3D model.

### Spatial profile of delivery (Fig 2)

**Fig 2:**
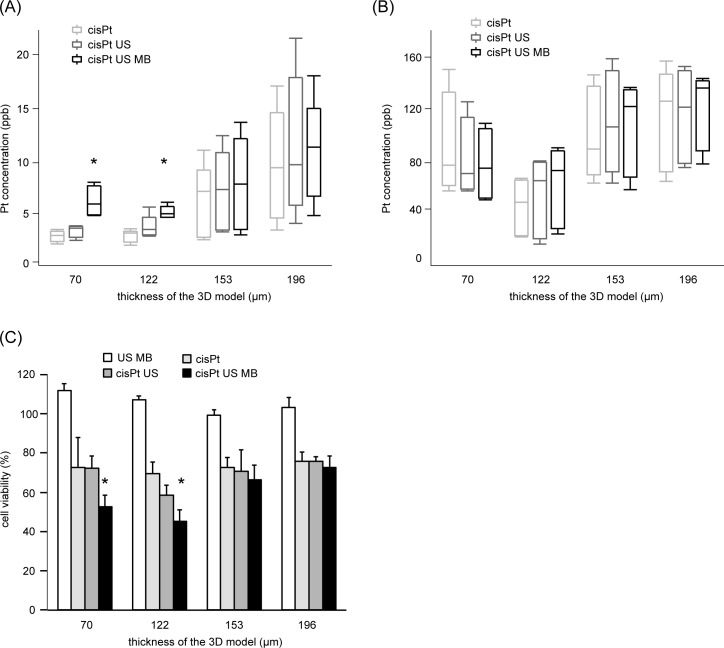
Cisplatin delivery by US-triggered microbubble cavitation to UM-UC-3 cells in the 3D culture at different thickness. UM-UC-3 cells in the 3D culture were exposed to ultrasound for 1 min with 6 μg/mL cisplatin and 190 μL Sonazoid^TM^ microbubbles. (A) Intracellular platinum concentration, (B) extracellular platinum concentration. Cells were harvested immediately after sonication, and the cell pellet and the dissolved collagen gel were digested in 60% HNO_3_. Platinum concentration was measured by ICP-MS. Data are shown in box plot (n = 5). (C) The cell viability. The cells were cultured for 4 days in cisplatin-free medium after the sonication. Cell viability was evaluated by the trypan-blue dye exclusion test. Data represent mean + standard deviation (n = 4). * indicates *p* < 0.05. cisPt, cisplatin; US, ultrasound; US MB, US-triggered microbubble cavitation.

Ultrasound-triggered microbubble cavitation increased the intracellular Pt concentration at the thickness of 70 and 122 μm (Fig 2A). However, the intracellular Pt concentration did not increase at the thickness of 153 or 196 μm (Fig 2A). The extracellular Pt concentration did not differ among treatments at any thickness of the 3D model (Fig 2B). The cell viability of the cisplatin and cavitation was significantly lower than other treatments at the thickness of 70 and 122 μm (Fig 2C).

### Temporal profile of delivery (Fig 3)

**Fig 3:**
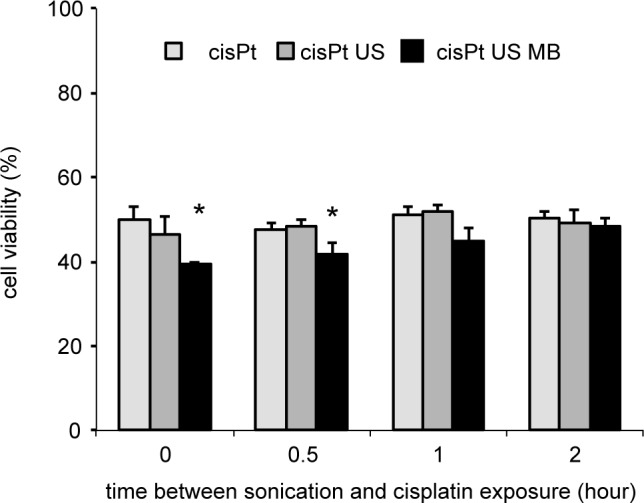
The viability of UM-UC-3 cells at different intervals between sonication and cisplatin exposure. UM-UC-3 cells in the 3D culture were exposed to ultrasound for 1 min with 190 µL Sonazoid^TM^ microbubbles. Cisplatin was added to the 3D model 0, 0.5, 1, or 2 hours after sonication. The cells were cultured for 4 days in cisplatin-free medium. Cell viability was evaluated by the trypan-blue dye exclusion test. Data represent mean + standard deviation (n = 4). * indicates *p* < 0.05. cisPt, cisplatin; US, ultrasound; US MB, US-triggered microbubble cavitation.

In order to decide the optimal intervals of treatment repetition, cells in the 3D model were exposed to ultrasound-triggered microbubble cavitation without cisplatin. Thereafter, cisplatin was added at the different time points. The cell viability significantly decreased when cells were exposed to cisplatin 0, 0.5 hr after sonication (Fig 3; p < 0.05).

### Multiple treatments (Fig 4)

Based on the spatial and temporal profile of cisplatin delivery by the single session of US-triggered microbubble cavitation, the feasibility of multiple treatments was assessed. Cells in the 3D model at the thickness of 196 µm were exposed to ultrasound three times at 30 min intervals. Triple US-triggered microbubble cavitation enhanced the cytotoxic effect of cisplatin at the thickness of 196 µm.

**Fig 4:**
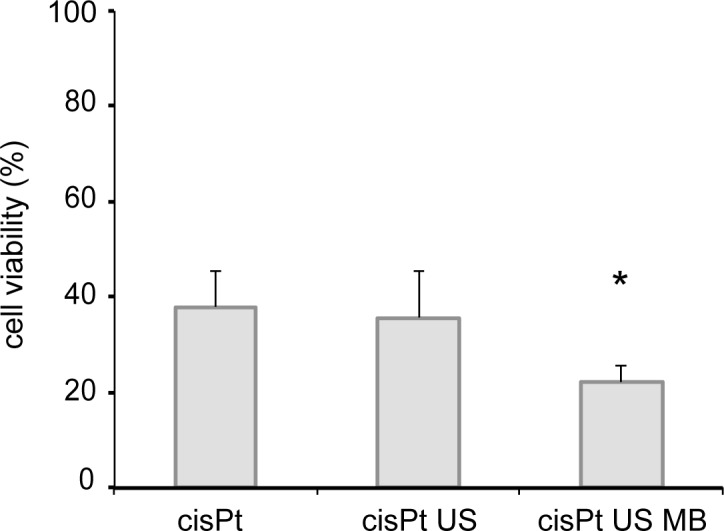
Cell viability of UM-UC-3 after the triple treatments. UM-UC-3 cells in the 3D model was exposed to ultrasound three times in the presence of 6 µg/mL cisplatin and 190 µL Sonazoid^TM^ microbubbles at 30 min intervals. The cells were cultured for 4 days in cisplatin-free medium after the treatment. Cell viability was evaluated by the trypan-blue dye exclusion test. Data represent mean + standard deviation (n = 3). * indicates *p* < 0.05. cisPt, cisplatin; US, ultrasound; MBUS, US-triggered microbubble cavitation.

## Discussion

The rationale for US-assisted intravesical chemotherapy in NIMBC is to achieve higher intracellular drug concentrations at the sonicated area than those achieved in the conventional intravesical therapy. Our results suggest that single treatment of US-triggered microbubble cavitation accomplished both the intracellular delivery and the enhanced cytotoxic effect of cisplatin in a scaffold-based 3D culture. Previous studies showed that US-triggered microbubble cavitation achieves the intracellular delivery of chemotherapeutic agents [25] and enhances the cytotoxic effect of the agents in monolayer culture or cell suspension [26–28]. Efficient drug penetration in tumor tissue and intracellular delivery are important goals in the development of US-assisted intravesical chemotherapy. It would be beneficial to quantitatively and objectively assess intracellular delivery and penetration characteristics of chemotherapeutic agents using predictive *in vitro* models. Moreover, 3D cultures can mimic the tumor microenvironment more precisely than conventional culture methods [29]. Our results may bridge the gap between the conventional culture methods and *in vivo* experiments.

The effective thickness of delivery in this study is comparable to the diffusion distance of choromophores into collagen-based structures by ultrasound with/without microbubbles [18,30]. It was reported that 1–2 mm depth of tumor invasion into lamina propria was a negative prognostic factor of non-muscle invasive bladder cancer [31,32]. Single treatment of US-mediated intravesical chemotherapy may not be able to treat even a superficial tumor. However, the advantage of US-triggered microbubble cavitation is the minimal invasiveness of the technology. Our results show that repetition of microbubble replenishment and US exposure enhanced the cytotoxic effect of cisplatin in the thicker 3D model. Combined with replenishment of microbubbles in bladder, US-assisted intravesical chemotherapy has a potential to treat thicker tumor in the manner of a peeler, i.e. a few layers by each treatment. In addition to the repetition of the treatment, the use of targeted microbubbles may be an alternative tool for improving the efficacy of US-assisted intravesical chemotherapy. Non-targeted microbubbles, such as clinical ultrasound contrast agents, may be suspended in bladder and rise up to bladder wall with the terminal velocity demined by their size. Sonazoid™ microbubbles were distributed in the medium in the present setup, which may attenuate US waves. Targeted-microbubbles facilitated microbubble attachment to tumor cells and induced sonoporation [33]. Repeated US-triggered microbubble cavitation with targeted microbubbles is promising for improving the efficacy of treatment.

Neither US nor US-triggered microbubble cavitation affected on extracellular Pt concentration at any thickness of the 3D model in this study. Cisplatin might rapidly diffuse into the whole gel even without ultrasound because its molecular size is small [34]. The diffusion is regulated by the diffusion coefficient based on the Stokes-Einstein equation; Diffusion coefficient [m^2^/s] = *k*T/6πηr (where *k* is the Boltzmann’s constant, T is temperature in K, η is the viscosity of the solvent, r is the radius of the particle). The calculated diffusion distance of cisplatin in water during the delivery period (5 min) is 639 µm. Even though we did not measure the viscosity of 3 mg/mL collagen gel, the diffusion distance in collagen gel is similar to that of in water [35]. Therefore, cisplatin might thoroughly diffuse into the whole gels. Meanwhile, cellular uptake of cisplatin is mainly regulated by transporters and partially by diffusion [36,37]. Ultrasound-triggered microbubble cavitation may increase cisplatin uptake by cells that located at superficial layers. Effects of a collapsing microbubble reduce rapidly with increasing the distant of microbubble from the cell membrane [38]. In addition to the cells adjacent to microbubbles, US-triggered microbubble cavitation may have a potential to enhance cisplatin uptake by cells that are moderately distant from microbubbles. Acoustic radiation force is one of the mechanisms of drug delivery using cavitation [39]. Although the acoustic pressure of this study was relatively low, addition of microbubbles may enhance the acoustic radiation force. Radiation force is given as follows; Radiation force = 2αI/c, in which α is the constant of absorption; I is the ultrasound intensity; c is the velocity of the sound wave. Besides the conventional mechanisms such as pore formation and endocytosis [39,40], it was reported that the stress on the cell membrane increases cisplatin uptake via transporters [41,42].

The duration of increased cell membrane permeability after US-triggered microbubble cavitation has been measured using impermeable chromophores and was in the order of seconds, minutes, and hours [10–13]. A recent comprehensive study demonstrated that the duration of increased cell membrane permeability was 1–3 hours despite different cell lines or US pressures [13]. Although we did not measure the intracellular Pt concentration, it seemed that effects of US-triggered microbubble cavitation continued for an approximately 1 hour in the present study. It must be noted that assessment of the exact mechanism of the cell membrane permeability by cavitation is not the primary object of this study, but that we intend to illustrate spatial and temporal profile of cisplatin delivery using the bladder cancer mimicking in vitro model.

In conclusion, it is shown that US-triggered microbubble cavitation is a promising tool for the intravesical chemotherapy in superficial bladder cancer. Single treatment of US-triggered microbubble cavitation increased the intracellular cisplatin concentration at the superficial region of the 3D model. Repetition of US-triggered microbubble cavitation may increase the efficacy of the treatment, and the interval of the repetition would be determined by the temporal profile of delivery.

## Supporting information

S1 Fig Overview of the experimental protocols. **(**A) Platinum quantification and cell viability assessment with different thickness of the 3D model. (B) Effect of an interval between sonication and cisplatin exposure on the cell viability. (C) Triple treatment of US-triggered microbubble cavitation.
(EPS)

S2 Fig Fluorescent microscopic images of UM-UC-3 cells in the 3D model. The thickness of each image was (A) 74, (B) 100, (B) 140, and (B) 182 µm, respectively. After culturing for 3 days, cells were stained with 2 µM Calcein-AM. Z-stuck images were taken with a confocal fluorescent microscopy.
(EPS)

S1 File Schematic outline of the sample pretreatment procedures for platinum quantification by ICP-MS. 3D, three-dimensional; MEM-alfa, alfa Minimum Essential Medium; PBS, phosphate buffered solution; HNO_3_, nitric acid; UPW, ultra-purified water; ICP-MS, inductively coupled plasma mass spectrometry.
(DOCX)
