# Supplementary material for: Spatial and temporal profile of cisplatin delivery by ultrasound-assisted intravesical chemotherapy in a bladder cancer model
Source: PLoS One. 2017 Nov 30;12(11):e0188093. doi: 10.1371/journal.pone.0188093 (PMC5708802; doi:10.1371/journal.pone.0188093)
Supplement: S1 File — 3D, three-dimensional; MEM-alfa, alfa Minimum Essential Medium; PBS, phosphate buffered solution; HNO3, nitric acid; UPW, ultra-purified water; ICP-MS, inductively coupled plasma mass spectrometry. (DOCX) [file pone.0188093.s003.docx]

Dissolved collagen gel

1. Take an aliquot of the supernatant
   1. 100 µL in 50 and 100 µL gel
   2. 50 or 100 µL in150 µL gel
   3. 25 or 50 µL in 200 µL gel
2. Hydrolyze with 60% HNO_3_
   1. 0.9 mL for 1.1
   2. 0.95 or 0.9 mL for 1.2
   3. 0.975 or 0.95 mL for 1.3
3. Dilute with 9 mL of UPW

Cell pellet

1. Add 1 mL of 60% HNO_3_
2. Dilute with 9 mL of UPW

3D culture (50, 100, 150, 200 µL)

1. Add 1 mL MEM-alfa containing 0.02% collagenase
2. Centrifuge
3. Transfer 0.8 mL of supernatant
4. Wash sediment twice with PBS

platinum analysis by ICP-MS

Standard curve for cell pellet; 1, 2.5, 5, 10, 25, 50 ppt

Standard curve for gel; 5, 10, 25, 50, 100 ppt
